# Resilience and job satisfaction among out‐of‐hospital emergency medical service professionals: A cross‐sectional multi‐centric study

**DOI:** 10.1111/jonm.13645

**Published:** 2022-06-07

**Authors:** Susana Mantas‐Jiménez, Maria Teresa Lluch‐Canut, Juan Roldán‐Merino, Glòria Reig‐Garcia, Dolors Juvinyà‐Canal

**Affiliations:** ^1^ University of Girona (Girona) Girona Spain; ^2^ Department of Psychosocial and Mental Health, School of Nursing, Faculty of Medicine and Health Sciences University of Barcelona Barcelona Spain; ^3^ Research Group GEIMAC (Consolidated Group 2014‐1139: Group of Studies of Invarianza of the Instruments of Measurement and Analysis of Change in the Social and Health Areas) (Barcelona), Research Group GIRISAME (International Researchers Group of Mental Health Nursing Care) Madrid Spain; ^4^ Research Group GEIMAC (Group Consolidat 2014‐1139: Grupo de Estudios de Invarianza de los Instrumentos de Medida y Análisis del Cambio en los Ámbitos Social y de la Salud) Barcelona Spain; ^5^ Research Group GIRISAME (International Researchers Group of Mental Health Nursing Care) Barcelona Spain; ^6^ Campus Docent Sant Joan de Déu‐Fundació Privada, School of Nursing University of Barcelona Barcelona Spain; ^7^ Department of Health Promotion University of Girona (Girona) Girona Spain

**Keywords:** emergency medical services, health care worker, job satisfaction, resilience

## Abstract

**Aim:**

We aim to describe the relationship between job satisfaction and compare levels of resilience among out‐of‐hospital emergency medical service professionals.

**Background:**

The study of the impact of the working environment on health professionals has raised great interest. Job‐related variables and resilience can be a protective factor against stressful and demanding events at work.

**Methods:**

A cross‐sectional survey comprising sociodemographic and job‐related variables was conducted among 406 workers (doctors, nurses, psychologists, and ambulance technicians) from the out‐of‐hospital emergency medical system in Spain. Resilience was self‐reported using the Connor‐Davidson Resilience Scale.

**Results:**

Nursing professionals were less resilient compared with ambulance technicians (score difference 1.709, *p* = .008). As age increased, resilience was lower (*r* = −.118). Professionals with higher resilience scores were more satisfied in their work (OR = 1.06, 95% CI: 1.02–1.11), and professionals with higher psychological strength, gained from working with other colleagues, also showed greater job satisfaction (OR = 5.47, 95% CI: 2.55–11.73).

**Conclusion:**

There was a positive association between resilience, job satisfaction and collaborative work. Professionals with greater psychological strength, gained from working with other colleagues, also showed higher levels of job satisfaction.

**Implications for Nursing Management:**

Managers can use these results to influence the work environment to enhance job satisfaction and hence improve the resilience of the out‐of‐hospital emergency health care professionals.

## 1 INTRODUCTION

Health is a resource that enables individuals to reach their full potential and contribute to the overall development of society. Health in work‐related contexts implies the adoption of an approach based on the positive perspective, which seeks a balance between preventive actions and actions that improve the health of individuals and communities (Cassetti et al., 2019). Among these actions is the capacity for resilience, understood as a set of intrinsic factors that characterize all individuals and that is involved in the process of overcoming adversity (Eakman et al., 2019; Ramirez‐Granizo et al., 2020). Health in work contexts is composed of multiple factors, including resilience (Delgado et al., 2020; Feng et al., 2017). Resilient people have positive emotions in stressful situations and maintain a great interest in everything that happens in their lives (Fredrickson et al., 2020).

Health professionals are a fundamental asset of health systems (World Health Organization, 2020). Health in work‐related contexts allows people to face their professional challenges, facilitating their adaptation to stressful working conditions, management of emotions, the development of coping strategies, improvement of well‐being and their professional growth (Foster et al., 2018). Likewise, other authors have proposed the term ‘professional resilience’ to refer to the ability to adapt to changing circumstances, even when threatening changes occur (Sommer et al., 2016). The growing research on the study of resilience among health care professionals highlights the need for more evidence on the subject, specifically on the study of resilience among out‐of‐hospital emergency medical care professionals.

Out‐of‐hospital emergency medical care professionals provide care to people in health emergencies, often dealing with difficult cases in complex and traumatic environments (Shakespeare‐Finch & Daley, 2017). They include nurses, emergency technicians and medical staff and are responsible for providing health care at the individual and/or collective level, in a critical situation. In addition, nursing professionals are also present in coordination centres and in service management. Service management integrates the work of all health care workers and is responsible for clinical practice and quality of care to ensure patient and professional safety. Their contributions are vital to the planning of organizational and emergency responses in a critical situation. Due to the nature of their work, out‐of‐hospital emergency medical care professionals are at a higher risk of developing mental health disorders than the general population. For example, 74% of emergency medical care professionals in Iran reported moderate levels of stress (Seyedjavadi et al., 2014). Preserving and improving the emotional health of out‐of‐hospital emergency health care workers is critical not only for the professionals themselves but also for the community to which they provide health care (Halpern et al., 2009).

Resilience is a multidimensional characteristic that refers to the personal qualities that enable an individual to thrive in the face of adversity (Charney, 2004; Connor & Davidson, 2003; Davidson et al., 2005). It has also been described as a context‐dependent, individual dynamic phenomenon capable of improving the quality of clinical services by reducing work‐related stress and thereby increasing well‐being (Flanagan & Flanagan, 2002). Resilience enables individuals to cope with their professional challenges (Grant & Kinman, 2014; McDonald et al., 2016), facilitating adaptation to stressful conditions, emotion management, the development of coping strategies, improved well‐being and personal growth (Stephens, 2013). Conceptual models of psychological resilience in nurses have been proposed, but most focus on individual characteristics (Foureur et al., 2013; Gillespie et al., 2007; Rees et al., 2015) rather than work‐related factors. For this reason, it is important to study how resilience processes develop and what are the factors that can influence the work of individuals exposed to adverse conditions (Ribeiro et al., 2011). There are several factors that can improve the level of resilience of out‐of‐hospital emergency health care professionals and promote service quality. Among these factors, scene safety and security, decision‐making, self‐efficacy and religious support were found to be effective in improving resilience and quality of care in emergency professionals (Froutan et al., 2015). In another study, a meta‐analysis was conducted to determine how workplace interventions influence nurses' job satisfaction. The interventions were primarily educational and were found to be effective in improving job satisfaction (Niskala et al., 2020). This study highlighted the need to consider the implementation of effective interventions to improve job satisfaction among health care professionals. Workers with higher levels of resilience score and higher score in creative thinking have higher job satisfaction and cope better with adversity (Golparvar et al., 2013).

Regarding the evaluation of the construct, the following questionnaires can be highlighted: Baruth Protective Factors Inventory – BPFI (Baruth & Carroll, 2002); Brief‐Resilient Copping Scale ‐ BRCS (Sinclair & Wallston, 2004); Adolescent Resilience Scale – ARS (Oshio et al., 2003); Resilience Scale for Adults (Friborg et al., 2003) and the Connor & Davidson Resilience Scale – CD‐RISC (Connor & Davidson, 2003). The latest has been shown to be a valid and reliable instrument for measuring individual psychological resilience. It has been used in the general population and in health care workers.

This study of the relationship between resilience, sociodemographic and job‐related variables in out‐of‐hospital emergency service professionals can help health service managers to understand the level of job satisfaction and coping mechanisms among these professionals. We aim to describe the relationship between job satisfaction and compare levels of resilience among out‐of‐hospital emergency medical service professionals in the health region of Catalonia, Spain. The questions that guided this study were as follows: (i) Are there any differences among professionals in terms of level of resilience? (ii) Do sociodemographic and job‐related variables influence job satisfaction and, consequently, the resilience of out‐of‐hospital emergency medical services professionals? And (iii) Can job satisfaction increase the level of resilience in out‐of‐hospital emergency medical services professionals?

## 2 METHODS

### 2.1 Study design

A cross‐sectional and correlational study design was used to investigate the relationship between sociodemographic variables—sex, age, household members and dependent family members—and job‐related variables—professional category, type of contract, work shift, collaboration with other institutions and job satisfaction and resilience in out‐of‐hospital emergency medical care professionals.

### 2.2 Participants

The population consisted of 493 out‐of‐hospital emergency health care professionals from the health region of Catalonia, Spain, belonging to the following professional categories: doctors, nurses, psychologists and ambulance technicians, from eight emergency health care bases. Out‐of‐hospital emergency medical services are publicly managed, attached to the Department of Health of the Generalitat de Catalunya, Spain. They are specifically designed, staffed and equipped for the emergency care of patients. This service is provided by a team of professionals, whose main objective is to respond to out‐of‐hospital health emergencies and emergencies quickly, efficiently and with the highest level of quality, 24 h a day, 365 days a year. The sample size was calculated to detect an effect size of Cohen's between two groups of 0.30, with a power of 80% and a risk α of 5% (Cohen, 1988). A minimum of 175 participants was required in each group. Professionals who had been in their current job for more than a year and were willing to voluntarily consent to participating in the study met the inclusion criteria. Health care professionals on refresher courses or internships were excluded. A total of 406 health care professionals finally took part.

### 2.3 Data collection

The care managers of each base participating in the study were contacted, and a presentation of the project was scheduled at each one. The presentation included an explanation of the research objectives, the measurement instruments, informed consent and the procedure for distributing the data collection booklet in which the participants were informed of the voluntary nature of participation, anonymity and confidentiality of the data. On the same day as the study was presented, a member of the research team delivered the questionnaires and an envelope to the care manager. The same procedure took place at all eight bases. The participants were given 30 days to answer the questionnaires. Once the questionnaires had been answered, they were placed in the sealed envelope and returned to the manager of each base, who in turn kept them in a sealed box until collection. Data collection was carried out between September and October 2017, by a member of the research team.

### 2.4 Measurements

A self‐completed form containing the following four items was used to measure the sociodemographic variables: *Age*; *Sex*; *Household members*: I live alone (0), with family/partner (1); and *Dependent family members*: I have dependents, partner/children/other (1), I do not have dependents, partner/children/other (0). The sociodemographic variables of the study sample are shown in Table 1. Participants self‐completed a further five items to measure the job‐related variables: *Professional category*: doctor (1), nurse (2), ambulance technician (3), psychologist (4); *Type of contract*: permanent (1), temporary (2); *Work shift*: 24‐h shifts: the professional works 24 h at a time (a full day) and then has a 24‐h break (1); rotating shifts: the professional works day or night shifts, which are shorter. The duration depends on the care provided to the population (2); *Job satisfaction* understood as the degree of overall job satisfaction: In relation to health care work, what is your overall degree of job satisfaction?: not at all satisfied (1), not very satisfied (2), quite satisfied (3), very satisfied, (4) totally satisfied (5); and last, *Collaboration with other institutions*, measuring the degree to which collaborative work with other institutions (justice, law enforcement authorities) gave the health care professionals strength in the positive sense of the term: In general, does working with/collaborating with colleagues from other institutions give me strength?: not at all (1), rarely (2), sometimes (3), often (4), always (5).

**TABLE 1:** Sociodemographic variables

Characteristics	Explanation
Age	
Sex	
Women	
Men	
Number of persons in household	The term ‘household’ includes all the people occupying a housing unit. Includes the family householder and all other people in the living quarters who are related to the householder by birth, marriage, or adoption.
Living alone (0)	
Living with partner/family (1)	
Dependent family members	A dependent family member is a person who relies on someone else for financial support and can include children or other relatives.
Yes I have dependents, partner/children/other (1)	
No, I do not have dependents, partner/children/other (0)	

Resilience was measured using the Connor‐Davidson Resilience Scale (CD‐RISC), designed by Connor and Davidson (2003). The CD‐RISC scale was developed for clinical practice as a measure of resilience. The original scale is made up of 25 Likert‐type items where high scores indicate a higher level of resilience. In this study, we used the version adapted to Spanish, the 10‐item CD‐RISC scale (P1, P2, P3, P4, P5, P6, P7, P8, P9 and P10), validated in a population of non‐institutionalized adults aged between 60 and 75 (Serrano‐Parra, 2012). The 10‐item CD‐RISC scale in Spanish had satisfactory content, concurrent and discriminant validity, and Cronbach's α of .81, indicating a high internal consistency reliability (Serrano‐Parra, 2012). The scale was self‐administered, and the 10 items were organized in a one‐dimensional, summative Likert‐type scale: *not at all* (1), *rarely* (2), *sometimes* (3), *often* (4), *almost always* (5). The scale ranged between 10 and 50 points where the higher the score, the greater the resilience.

### 2.5 Statistical analysis

The data were analysed using SPSS 22.0 version (IBM CORP., Armonk, NY). Descriptive statistics were used to describe the levels of resilience and sociodemographic and job‐related variables. Central tendency values were obtained for the quantitative variables, and the frequency and percentage calculated for each of the categories to describe the qualitative variables. Student's *t*‐tests and one‐way ANOVA were performed to compare mean scores in resilience with the following variables: household members, professional category, type of contract, work shift, job satisfaction, and collaboration with other institutions. Post hoc comparisons were performed for statistically significant associations using the Bonferroni test. The relationship with age was analysed using the Pearson correlation coefficient. Bonferroni correlation for multiple comparisons was performed for univariate analysis. A multivariate logistic regression analysis was performed to generate the odds ratio for being more satisfied with work and to analysed the relationship with the total resilience score. In this model, the job satisfaction variable was categorized into two categories (less satisfied = not at all satisfied/not very satisfied/quite satisfied and more satisfied = very satisfied/fully satisfied). We adjusted for age, sex, professional category (doctors, nurses and ambulance technicians) and CW = gives me strength (less strength = not at all/rarely and more strength = sometimes, often and always). The odds ratio and its 95% confidence interval were calculated for univariate and multivariate analyses. The reliability of the CD‐RISC scale was also analysed in this sample using Cronbach's alpha coefficient for the total scale. Probability values (*p*‐values) of less than .05 were considered significant.

## 3 RESULTS

A total of 406 professionals from the out‐of‐hospital emergency health care system participated in the study. The majority of participants were men (66.3%), and the average age was 38.2 years (SD 7.5). The majority lived with their partner and/or family at the time of the study (83.5%) and had dependents (63.3%). Regarding the professional category, 49.3% of the sample studied were ambulance technicians, 39.2% were nurses, 10.1% were doctors and 1% were psychologists. Because the percentage of psychologists employed by the out‐of‐hospital emergency health system at the time of the study was very low, this professional category was not included in the subsequent statistical analyses. A total of 88.4% of participants had a permanent contract, and 39.4% did rotating shift work. The sociodemographic and job‐related variables of the study sample and levels of resilience in relation to the study variables are shown in Table 2. Significant differences were obtained in the professional category. In post hoc comparisons, nurses showed lower resilience compared with ambulance technicians (mean difference 1.709; *p* = .008; Cohen's effect d = 0.33). Doctors also showed lower resilience compared with ambulance technicians, although the differences were not statistically significant (mean difference 2.177; *p* = .067; Cohen's effect d = 0.43). As age increased, resilience was lower (*r* = −.118; *p* = .018), and those who were more resilient were workers who reported greater job satisfaction and those who claimed that working with other co‐workers gave them greater strength, although the differences were not statistically significant.

**TABLE 2:** Relation between sociodemographic and employment variables and the Connor‐Davidson Resilience Scale (CD‐RISC) of the study sample (*n* = 406)

Characteristics	*n*	%	Mean	SD	*p*
Sex					
Women	134	33.0	40.2	5.0	.362^a^
Men	269	66.3	40.7	5.0	
Number of persons in household					
Living alone	65	16.0	40.8	5.4	.677^a^
Living with partner/family	339	83.5	40.5	4.9	
Dependent family members					
Yes	257	63.3	40.4	5.1	.268^a^
No	139	34.2	41.0	4.8	
Professional category					
Doctor	41	10.1	39.2	4.8	.004^b^
Nurse	159	39.2	39.7	4.8	
Ambulance technician	200	49.3	41.4	5.1	
Psychologist	4	1.0	40.2	2.5	
Type of contract					
Permanent	359	88.4	40.5	5.1	.686^a^
Temporary	39	9.6	40.8	3.7	
Work shift					
24‐h	139	34.2	41.2	4.9	.057^b^
Rotating shifts	160	39.4	39.8	5.2	
Dont know/no answer	107	26.4	40.8	4.8	
Degree of job satisfaction					
Not at all satisfied	3	0.7	34.0	13.0	.061^b^
Not very satisfied	20	4.9	38.2	8.5	
Quite satisfied	141	34.7	39.9	4.8	
Very satisfied	190	46.8	40.9	4.5	
Totally satisfied	39	9.6	42.3	3.9	
CW ‘gives me strength’					
Not at all	14	3.4	37.7	9.0	.020^b^
Rarely	28	6.9	41.2	4.3	
Sometimes	125	30.8	39.6	4.7	
Often	163	40.1	40.8	4.6	
Always	64	15.8	42.1	5.1	

Abbreviations: CW, ‘collaborative work’, p, level of significance = 0.005 (Bonferroni correction); SD, standard deviation.

^a^ Student's *t*‐test and Fisher's exact test.

^b^ ANOVA.

The overall resilience levels of the professionals in the out‐of‐hospital emergency medical system and the mean scores per item are shown in Table 3. The 10 items that make up the CD‐RISC scale are presented in English and Spanish versions. In detail, the item that obtained the highest mean score was item P1 ‘I am able to adapt when changes occur’ (mean = 4.54, SD = 0.71), whereas the item obtaining the lowest mean value was P10 ‘In facing life's problems and difficulties, sometimes you have to act on a hunch without knowing why’ (mean = 3.25, SD = 1.01). According to the results, the mean total score on the resilience scale for the participants in the study was 40.6 (SD 5.0), with minimum and maximum scores of 19 and 50 points, respectively (range = 31).

**TABLE 3:** Descriptive statistics of Connor‐Davidson Resilience Scale (CD‐RISC) items

Summarized item contents		Mean	SD	% floor	% ceiling
P1	I am able to adapt when changes occur *Soy capaz de adaptarme cuando ocurren cambios*	4.54	.711	1.0	63.1
P2	I can deal with whatever comes my way *Puedo enfrentarme a cualquier cosa que se me presente*	4.28	.773	1.0	44.1
P3	I try to see the humorous side of things when I am faced with problems *Intento ver el lado divertido de las cosas cuando me enfrento a los problemas*	3.90	.992	2.7	32.5
P4	Having to cope with stress can make me stronger *Enfrentarme a los problemas puede hacerme más fuerte*	4.20	.833	.5	43.1
P5	I tend to bounce back after illness, injury or other hardships *Tengo tendencia a recuperarme pronto tras enfermedades, heridas o adversidades*	4.38	.798	.7	53.9
P6	I believe I can achieve my goals, even if there are obstacles *Creo que puedo lograr mis objetivos incluso si hay obstáculos*	4.17	.796	1.0	37.4
P7	I stay focused and think clearly under pressure *Bajo presión me centro y pienso claramente*	4.01	.875	1.0	33.0
P8	I am not easily discouraged by failure *No me desanimo fácilmente con el fracaso*	3.65	1.048	3.2	22.9
P9	I think of myself as a strong person when dealing with lifes challenges and difficulties *Creo que soy una persona fuerte cuando me enfrento a los retos y dificultades de la Vida*	4.22	.814	1.0	42.6
P10	In dealing with lifes problems and difficulties, sometimes you have to act on a hunch, without knowing why *Al enfrentarme a los problemas y dificultades de la Vida, a veces actúo por un presentimiento sin saber porqué*	3.25	1.01	3.7	11.3
	Total CD‐RISC	40.6	5.0		

Abbreviations: P1 to P10, 10 item CD‐RISC; SD, standard deviation.

In the multivariate analysis, the final model showed that the variables related to the degree of job satisfaction were resilience scores and the strength gained from working with other colleagues (Table 4). The professionals with higher resilience scores were more satisfied in their work (OR = 1.06; 95% CI: 1.02–1.11). Health workers with higher strength gained from working with other colleagues also reported greater job satisfaction (OR = 5.47; 95% CI: 2.55–11.73). Furthermore, in this study, the reliability of the CD‐RISC scale was analysed. Cronbach's α for internal consistency was 0.78, which is considered adequate (Nunnally & Bernstein, 1994).

**TABLE 4:** Multiple logistic regression model to analyse variables associated with job satisfaction (*n* = 384)

Characteristics	Analysis univariate			Analysis multivariate		
	OR	95% CI	*p*	OR adjusted	95% CI	*p*
CD‐RISC	1.06	1.02–1.11	.003	1.06	1.02–1.11	.005
Age	1.00	0.97–1.03	.985	1.00	0.97–1.03	. 794
Sex						
Men	1			1		
Women	0.67	0.44–1.03	.070	0.77	0.48–1.23	.273
Number of persons in household						
Living alone	1					
Living with partner/family	1.18	0.68–2.04	.546			
Dependent family members						
Yes	1					
No	0.84	0.55–1.28	.426			
Professional category						
Doctor	1			1		
Nurse	1.49	0.75–2.98	.251	1.41	0.67–2.97	.363
Ambulance technician	2.05	1.03–4.04	.039	1.60	0.75–3.42	.226
Type of contract						
Temporary	1					
Permanent	0.48	0.22–1.02	.057			
CW ‘gives me strength’						
Not at all/rarely	1			1		
Sometimes/often/always	5.28	2.51–11.10	.0001	5.47	2.55–11.73	.0001

Abbreviations: CI, confidence interval; CD‐RISC, Connor‐Davidson Resilience Scale; CW, ‘collaborative work’, OR, odds ratio.

## 4 DISCUSSION

Our study was conducted to describe the relationship between job satisfaction and compare levels of resilience among out‐of‐hospital emergency medical service professionals. The professionals' resilience was moderate to high, with the highest mean observed in the ability to adapt to changing environments, which is important considering that the activity of out‐of‐hospital emergency medical services professionals is carried out mostly in unexpected situations. In another study, the resilience of health care professionals in emergency services was shown to be moderate to low (Sánchez‐Zaballos & Mosteiro‐Díaz, 2021). In our study, professionals with the professional category of nurses showed lower resilience compared to ambulance technicians, results that would be in line with other studies conducted with nurses working in public hospitals (Guo et al., 2017; Zou et al., 2016). The fact that ambulance technicians showed greater resilience may be due to the fact that ambulance technicians are the first to arrive at the scene of an emergency. Due to the variability of the emergencies they attend to, they are likely to develop more resilience, especially when the experience is more unpleasant. Future studies should consider analysing different professional groups to report on measures for improving levels of resilience among health professionals. For their part, Clark et al. (2021) found in their study on nurses in emergency services, that resilience was higher when job satisfaction was greater and as age increased. In our study, resilience was lower (*r* = −.118) as age increased. Along these lines, a study examining the fit of different equivalent measurement models for the factor structures of the Connor‐Davidson Resilience Scale (CD‐RISC) concluded that age was not a decisive variable (Pulido et al., 2020). Consequently, the study would have to be extended to older professionals to provide further evidence.

The variables related to resilience were the degree of job satisfaction and the psychological strength obtained when working with other colleagues. Coinciding with the results of this study, researchers have previously found an association between job satisfaction and resilience (Larrabee et al., 2010; Matos et al., 2010). Along the same lines, results with samples of professionals working in hospital settings showed that resilience is positively related to job satisfaction (Yang et al., 2017). In another study conducted with psychiatric nurses in Singapore, a positive and significant association was obtained between resilience and job satisfaction (Zheng et al., 2017). Hou et al. (2020) reported that job satisfaction and resilience have a significant influence on job performance. In his study, Hudgins (2016) reported significant relationships between resilience, job satisfaction and anticipated turnover. Zhao et al. (2021) also reported that resilience indirectly influences turnover intention through job satisfaction and social support.

Another study that investigated nurses' resilience (Öksüz et al., 2018) noted that the significant factors in their participants' resilience were perceived social support and job satisfaction, among other variables. A study on health professionals in emergency services (Sánchez‐Zaballos & Mosteiro‐Díaz, 2021) concluded that professional resilience is influenced by sociodemographic and occupational factors, all of which support our findings. Other studies (Kuokkanen et al., 2009; Teo et al., 2013) have found a positive association between organizational commitment and job satisfaction, highlighting those organizational changes that have a direct effect on the work environment in terms of empowerment and job satisfaction. These results are consistent with our study insofar as out‐of‐hospital emergency medical services professionals with greater strength gained from working with other colleagues also reported higher job satisfaction.

This study has some limitations. First, the exploratory, cross‐sectional design of the study limited our ability to infer causal relationships between contract type, work shift, job satisfaction, collaborative work with other institutions and resilience. The use of a longitudinal approach would be appropriate in future research to explain causal relationships and variables influencing long‐term resilience. Second, response bias could have affected the results, as the study was based on self‐completed measurements. Although Catalonia, Spain, is a large health care area, the sample might not reflect the perceptions of other out‐of‐hospital emergency health care professionals in other countries.

## 5 CONCLUSION

In our study, out‐of‐hospital emergency professionals had moderate to high levels of resilience. By professional category, nurses working in out‐of‐hospital emergency medical services were less resilient compared to ambulance technicians. Among the important findings, as age increased resilience was lower. Resilience was positively related to job satisfaction and psychological strength gained from working with colleagues. Professionals with greater psychological strength gained from working with colleagues also showed higher levels of job satisfaction.

## 6 IMPLICATIONS FOR NURSING MANAGEMENT

Our results indicate that there is a positive association between resilience, job satisfaction and collaborative work in out‐of‐hospital emergency medical service professionals. Considering that the work of these professionals is performed in changing and challenging environments, managers should take into account working conditions in order to influence the work environment and enhance job satisfaction and, therefore, improve the resilience of their staff.

## CONFLICT OF INTERESTS

The authors declare that they have no known competing financial interests or personal relationships that could have appeared to influence the work reported in this paper.

## ETHICS STATEMENT

Ethical approval was obtained from the Clinical Research Ethics Committee on Clinical Research of the Emergency Medical System (SEM). Also, participants were informed about the aim of the study before data collection, and their permissions were obtained. The principles defined in the Declaration of Helsinki were followed.

## AUTHOR CONTRIBUTIONS

SM and MTLL conceived and designed the study. JR and SM supervised the conduct of the data collection. JR and GR provided statistical advice on study design and analysed the data; SM, MTLL, GR and DJ drafted the manuscript, and all authors contributed substantially to its revision. MTLL takes responsibility for the paper as a whole.

## Data Availability

The data that support the findings of this study are available on request from the corresponding author. The data are not publicly available due to privacy or ethical restrictions.
